# A New Pronouncing Dictionary of Medicine

**Published:** 1895-06

**Authors:** 


A New Pronouncing Dictionary of Medicine: Being a voluminous and exhaustive hand-book of medical and scientific terminology, with phonetic pronunciation, accentuation, etymology, etc. By John M. Keating, M. D., LL. D., fellow of the College of Physicians of Philadelphia; vice-president of the American Pædiatric Society; ex-president of the Association of Life Insurance Medical Directors; formerly visiting obstetrician to the Philadelphia Hospital (Blockley), and lecturer on the diseases of women and children; consulting physician for the diseases of women, St. Agnes' Hospital, Philadelphia; gynæcologist to St. Joseph's Hospital, Philadelphia; editor *Cyclopædia of the Diseases of Children*, etc.; and Henry Hamilton, author of *A New Translation of Virgil's Æneid into English Rhyme*; co-author of *Saunder's Medical Lexicon*, etc. With the collaboration of J. Chalmers Da Costa, M. D., and Frederick A. Packard, M. D. With an appendix containing important tables of bacilli, micrococci, leucomaïnes, ptomaïnes; drugs and materials used in antiseptic surgery; poisons and their antidotes; weights and measures; thermometric scales; new officinal and unofficinal drugs, etc., etc. Prices: Cloth, $5.00 net; sheep, $6.00 net. W. B. Saunders, publisher, 925 Walnut Street, Philadelphia, Pa.

